# Modeling and Mathematical Investigation of Blood-Based Flow of Compressible Rate Type Fluid with Compressibility Effects in a Microchannel

**DOI:** 10.3390/mi13101750

**Published:** 2022-10-16

**Authors:** Kamel Guedri, Maha M. A. Lashin, Aamar Abbasi, Sami Ullah Khan, El Sayed Mohamed Tag-ElDin, Muhammad Ijaz Khan, Fozia Khalil, Ahmed M. Galal

**Affiliations:** 1 Mechanical Engineering Department, College of Engineering and Islamic Architecture, Umm Al-Qura University, P.O. Box 5555, Makkah 21955, Saudi Arabia; 2 College of Engineering, Princess Nourah Bint Abdulrahman University, Riyadh 11564, Saudi Arabia; 3 Department of Mathematics, University of Azad Jammu & Kashmir, Muzaffarabad 13100, Pakistan; 4 Department of Mathematics, COMSATS University Islamabad, Sahiwal 57000, Pakistan; 5 Faculty of Engineering and Technology, Future University in Egypt, New Cairo 11835, Egypt; 6 Department of Mathematics and Statistics, Riphah International University I-14, Islamabad 44000, Pakistan; 7 Mechanical Engineering Department, College of Engineering, Prince Sattam Bin Abdulaziz University, Wadi Addawaser 11991, Saudi Arabia; 8 Production Engineering and Mechanical Design Department, Faculty of Engineering, Mansoura University, Mansoura 35516, Egypt

**Keywords:** peristaltic flow, compressibility effects, acoustic wave, Maxwell fluid, microchannel

## Abstract

In this investigation, the compressibility effects are visualized on the flow of non-Newtonian fluid, which obeys the stress–strain relationship of an upper convected Maxwell model in a microchannel. The fundamental laws of momentum and mass conservation are used to formulate the problem. The governing nonlinear partial differential equations are reduced to a set of ordinary differential equations and solved with the help of the regular perturbation method assuming the amplitude ratio (wave amplitude/half width of channel) as a flow parameter. The axial component of velocity and flow rate is computed through numerical integration. Graphical results for the mean velocity perturbation function, net flow and axial velocity have been presented and discussed. It is concluded that the net flow rate and $D_{wall}$ increase in case of the linear Maxwell model, while they decrease in case of the convected Maxwell model. The compressibility parameter shows the opposite results for linear and upper convected Maxwell fluid.

## 1. Introduction

Peristalsis is one of the attractive flow mechanisms for material transport in various biological sciences; in particular, the peristaltic mechanism refers to the transport of urine from kidney to bladder, the chyme movement in the gastrointestinal tract, and the transport of spermatozoa in the ductus efferentes of the male reproductive tracts. Moreover, numerous biomedical and engineering equipment are designed on the principle of peristalsis. After the pioneering work of the first Latham [1], numerous researchers have evaluated peristaltic motion theoretically under different physical situations. Some motivational studies regarding the peristaltic motion follow: Narla et al. [2] discussed the transport of embryo in uterine. Ahmad et al. [3] highlighted the effects of wall compliance on the flow. Ali et al. [4] numerically investigated the motion of bio-rheological fluid. Reddy et al. [5] studied the peristaltic flow of coupled stress fluid. Tanveer et al. [6] studied some application of nanofluid. Javed et al. [7] discussed the peristaltic motion at moderate Reynolds numbers. Trapathi et al. [8] investigated the effects of applied electric field on the flow features. Hayat et al. [9] pointed out the effects of the chemical reaction. Javid et al. [10,11] studied the peristaltic motion in a complex wavy channel, and Prakash et al. [12] pointed out the applications of hybrid nanofluids.

The impact of ultrasonic radiations on the flow rate in a porous medium has wide applications in the oil industry. Chen et al. [13] were the first to investigate the significance of ultrasonic radiations configured by a tube. Cherskiy et al. [14] and Duhon [15] investigated applications of radioactive ultrasonic applications in flow rate experimentally and concluded that rate of flow increases throw narrow tubes. Geniev et al. [16] analyzed the enhancement in flow pattern based on the peristaltic mechanism. This investigation reveals that the ultrasonic radiation generated by the source induced a traveling wave confined by a porous tube. The mechanism proposed in reference [16] was discussed theoretically for a viscous fluid by Aarts and Ooms [17] in a porous medium represented by the set of many nonintersecting circular tubes. The governing Maxwell liquid relations have been used by Tsiklauri and Beresnev [18] to study the effects of relaxation time parameter on the net flow rate, and they concluded that as the relaxation time increases, the increase in the viscoelastic effects of the back flow regime induces an increase in the oscillation of net flow rate. Hayat et al. [19] used the constitutive equation of a linear Jeffrey model and concluded that the net flow rate has less oscillation than the linear Maxwell fluid model used by Tsiklauri and Beresnev. Mekheimer and Wahab [20] discussed the effects of slip parameter and compressibility on the back flow by considering the two annulus. The fluid transport induced by a surface acoustic wave in a microchannel was also discussed by Mekheimer and Wahab [21], and they also examined the effects of wall properties. Abbasi et al. [22] analyzed the compressibility effects on the peristaltic flow of viscoelastic fluid governed by the constitutive equation of an upper convected Maxwell fluid in an axisymmetric tube and concluded that the flow rate is greater in the upper convected case than in the linear Maxwell model. Abdelslam and Vafai [23] investigate the effects of compressibility on the peristaltic flow of electrically conducting Jeffrey fluid in a channel with permeable walls.

The flow in microchannels has become an area of widespread research interest due to applications in industry such as microelectromechanical systems, biomedical applications and micro-electric chip cooling. A microfluidic channel built in a biomicroelectromechanical structure is affected by environment noises such as oscillations, vibration or externally excited surface acoustic waves. Hajji et al. [24] simulated the kinetics of antigen–antibody binding in a microchannel. In another study, Hajji et al. [25] used a microfluidic biosensor for the detection of protein in a microchannel. Aissa et al. [26] numerically investigated the influence of wall slip on the gas flow in a microchannel. Recently, Hajji et al. [27] presented two-dimensional flow through a microchannel with contracting/expanding walls. Dehkordi et al. [28] discussed the effects of electric field on the flow of water-based ionic-oxide nanofluid through a microchannel and pointed out that the velocity, temperature and density of the nanofluid increases under the action of an external electric field. Ajili et al. [29] discussed microchannels as industrial equipment and numerically investigated the effects of burners’ heating load distribution in a hot-rolling preheating furnace. Asgari et al. [30] simulated the copper nano-channel with sphere barriers to study the flow of a water-based copper nanofluid. Farzinpour et al. [31] reported the heat transfer properties of ferro-nanofluid inside the microchannel. Some interesting work regarding the usage and application of microchannels has been recently reported by [32,33,34,35,36].

It is a well-known fact that the industrial and biological fluids such as blood are non-Newtonian in nature, and the rheology of these fluids is complex and cannot described by a single stress–strain relationship; at this end, several mathematical models have been presented and used [37,38,39,40,41] to discuss different flow and heat transfer characteristics. Refs. [42,43,44,45,46,47,48,49] highlight some important results in fluid flow versus dynamic surfaces. Recently, blood was studied using the particle-based techniques, models have built-in functions to describe compressibility effects, and the blood is transported through a peristaltic mechanism. Moreover, in all the studies mentioned above for compressible peristaltic flows, Newtonian or non-Newtonian linear constitutive equations are utilized. The human red blood cells have a large elastic and viscoelastic response. However, the use of a linear constitutive equation makes the applicability of the work to any real flow highly restricted. Therefore, there is a need to consider a nonlinear constitutive equation such as upper convected Maxwell to resemble more realistic physics.

In light of the above applications of flows in microchannels, this paper aims to discuss the flow of viscoelastic fluid. The constitutive equation of upper convected Maxwell fluid and another fluid model used by [50,51,52,53,54,55,56,57] having the properties both of elasticity and viscosity are used to describe the non-Newtonian rheology of the fluid. Along with the conservation laws, an equation of state is used to incorporate a variation in the density of the fluid. A comparative analysis has been carried out for the linear and convected mode. The paper is arranged in the following way: the mathematical form of governing equations and appropriate boundary conditions and their dimensionless formulation are given in Section 1. Under the implementations of the regular perturbation method, the obtained equations along with an analytical solution are presented in Section 2. Section 3 represents the graphical illustration and their discussion, while some main findings are summarized in Section 4.

## 2. Flow Model

**Figure 1:**
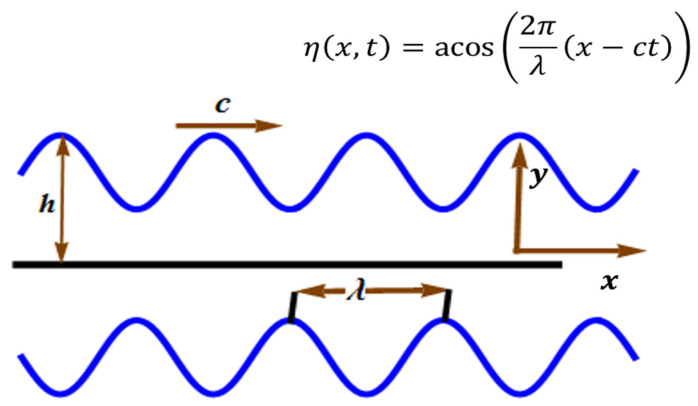
Geometry of the problem.

This model measures the flow of a compressible upper convected Maxwell liquid in a channel of length 2 h. The rectangular coordinates (x,y,z) are taken in such a way that that the x-axis is along the centerline of the channel and the y-axis is normal to it (see Figure 1). A sinusoidal wave of amplitude a causes a defamation of channel with flexible walls:

For the flow under consideration, the governing equations of motion are

(1) $$\frac{\partial \rho }{\partial t}+div\left(\rho V\right)=0,$$

(2) $$\rho \frac{dV}{dt}=-\nabla P+divS,$$

Equation (1) is the continuity equation that represents the mass balance, and Equation (2) represents the force balance on an arbitrary unit volume of fluid. The left-hand side of this equation is the inertial force per unit volume, whilst the right-hand side is the resultant surface force per unit volume. The units of each term on both sides are $kgm^{-2}s^{-2}$, where *V*, ρ,P are the velocity, density, and pressure of the fluid, respectively. For upper convected Maxwell fluid, the extra stress tensor S satisfies the constitutive relation [42,43]

(3) $$\left(1+\lambda_{1}\frac{D}{Dt}\right)S=\mu \left(\nabla V+{\left(\nabla V\right)}^{f}-\frac{2}{3}\nabla .V\right).$$

μ is the dynamic viscosity,$\;\lambda_{1}$ is the relaxation time, ∇V is the velocity gradient, f denotes the transpose, and D/Dt is the contravariant convected derivative which for any contravariant vector $b^{i}$ satisfies:

(4) $$\frac{Db^{i}}{Dt}=\frac{\partial b^{i}}{\partial t}+v^{r}b^{i},{\;}_{\;r}-v^{i},{\;}_{\;r}b^{r}.$$

It is important to mention that for a linear Maxwell fluid, the convected term vanishes. The no-slip boundary conditions satisfy the components of velocity, which are

(5) $$u\left(x,\pm h\pm \eta ,t\right)=0,\;v\left(x,\pm h\pm \eta ,t\right)=\pm \frac{\partial \eta }{\partial t}.$$

Here, u and v are velocity components in the axial and transverse direction, and η and –η are the vertical displacement of the upper and lower wall separately, which is expressed as

(6) $$\eta =acos\left(\frac{2\pi }{\lambda }\left(x-ct\right)\right),$$

a,  λ and c are the amplitude, wavelength and speed of the wave. The equation of state which describes the distinctive response liquid is:

(7) $$\frac{1}{\rho }\frac{\partial \rho }{\partial P}=K.$$

where K is the fluid compressibility. The solution of Equation (7) subject to the condition that $\rho_{0}$ denotes the constant density is expressed as

(8) $$\rho =\exp\left(K\left(P-P_{0}\right)\right).$$

Introducing the following dimensionless variables, the governing equations for the present analysis take the following form

$$\tilde{x}=\frac{x}{h},\tilde{y}=\frac{y}{h},\;\tilde{\eta }=\frac{\eta }{h},\;\tilde{u}=\frac{u}{c},\;\tilde{v}=\frac{v}{c},\;\tilde{\rho }=\frac{\rho }{\rho_{0}},\;\tilde{t}=\frac{ct}{h},\;\tilde{p}=\frac{p}{c^{2}\rho_{0\;}},{\tilde{p}}_{0}=\frac{p_{0}}{c^{2}\rho_{0\;}},\;{\tilde{\lambda }}_{1}=\frac{\lambda_{1}c}{h}$$

(9) $$\frac{\partial \rho }{\partial t}+u\frac{\partial \rho }{\partial x}+v\frac{\partial \rho }{\partial y}+\rho \left(\frac{\partial u}{\partial x}+\frac{\partial u}{\partial y}\right)=0,$$

(10) $$\left(1+\lambda_{1}\frac{D}{Dt}\right)\left(\rho \frac{\partial u}{\partial t}+\rho \left(u\frac{\partial u}{\partial x}+v\frac{\partial u}{\partial y}\right)\right)=\left(1+\lambda_{1}\frac{D}{Dt}\right)\frac{\partial p}{\partial x}+\frac{1}{Re}\left\{\nabla^{2}u+\frac{1}{3}\frac{\partial }{\partial x}\left(\nabla .V\right)\right\},$$

(11) $$\left(1+\lambda_{1}\frac{D}{Dt}\right)\left(\rho \frac{\partial v}{\partial t}+\rho \left(u\frac{\partial v}{\partial x}+v\frac{\partial v}{\partial y}\right)\right)=\left(1+\lambda_{1}\frac{D}{Dt}\right)\frac{\partial p}{\partial y}+\frac{1}{Re}\left\{\nabla^{2}v-\frac{2}{3}\frac{\partial }{\partial y}\left(\nabla .V\right)\right\}.$$

The dimensionless form of boundary conditions and Equation (8) can be written as

(12) $$u\left(x,\pm 1\pm \eta ,t\right)=0,\;v\left(x,\pm 1\pm \eta ,t\right)=\pm \frac{\partial \eta }{\partial t}.$$

(13) $$\rho =\exp\left(\chi \left(P-P_{0}\right)\right).$$

The dimensionless parameters α=(2πh/λ) denote the wave parameter, $Re=(\rho_{0}ch/\mu )$, Reynolds number, $\chi =\left(k\rho \rho_{0}c^{2}\right)$, and compressibility constant, η=εcos(α(x−t)), where ε=a/h is the amplitude ratio. Equation (13) is the dimensionless version of Equation (8), and therefore, it is independent of any units.

## 3. Solution of the Problem

Due to high nonlinearity, the governing equations subject to the boundary conditions cannot be solved with the help of any analytic method, so we assume that the amplitude ratio (wave amplitude/half width of channel) ε≪1 and apply the regular perturbation method to obtain the expressions of velocity components, pressure and the fluid density. For this purpose, following [17], we write for velocity, density and pressure as follows:

$$p=\sum_{i=1}^{\infty }\varepsilon^{i}p_{i}\left(x,y,t\right)u=\sum_{i=1}^{\infty }\varepsilon^{i}u_{i}\left(x,y,t\right),\;v=\sum_{i=1}^{\infty }\varepsilon^{i}v_{i}\left(x,y,t\right),\;\rho =1+\sum_{i=1}^{\infty }\varepsilon^{i}\rho_{i}\left(x,y,t\right)$$

Applying the similar procedure collecting the powers of ε in the form of $V_{1}$, $U_{1}$ and $P_{1}$ along with the boundary conditions

(14) $$V_{1}{}^{\prime }+\alpha iU_{1}=i\alpha P_{1},$$

(15) $$\gamma P_{1}-\frac{i}{\alpha }\left(U_{1}^{\prime \prime }-\beta^{2}U_{1}\right)=0,$$

(16) $$\gamma P_{1}^{\prime }-\left(V_{1}^{\prime \prime }-\beta^{2}V_{1}\right)=0,$$

(17) $$U_{1}\left(\pm 1\right)=0,V_{1}\left(\pm 1\right)=\mp \frac{i\alpha }{2},$$

where $\gamma =\left(1-i\alpha \lambda_{1}\right)Re-\frac{i\alpha \chi }{3}$, $\beta^{2}=\alpha^{2}-i\alpha \left(1-i\alpha \lambda_{1}\right)Re$. The solution of Equations (14)–(16) subject to Equation (17) is of the following form.

(18) $$U_{1}\left(y\right)=\frac{i\alpha }{\nu }c_{1}Cosh\nu y+\frac{i\beta }{\alpha }c_{2}Cosh\beta y,$$

(19) $$V_{1}\left(y\right)=c_{1}Sinh\nu y+c_{2}Sin\beta y,$$

(20) $$P_{1}\left(y\right)=\frac{\nu^{2}-\beta^{2}}{\nu \xi }c_{1}Cosh\nu y,$$

where $\xi =\frac{3Re-4i\alpha \chi }{3Re-i\alpha \chi }c_{1}$ and $c_{2}$ are complex constants which are obtained by Equation (17). Collecting the powers of $\varepsilon^{2}$ yields the following flow equations:

(21) $$V_{20}^{\prime }\left(y\right)=-\chi \frac{d}{dy}\left(P_{1}\bar{V_{1}}+\bar{P_{1}}V_{1}\right),$$

(22) $${U^{\prime \prime }}_{20}\left(y\right)=Ref\left(y\right),$$

(23) $$\begin{array}{c} f\left(y\right)=\frac{d}{dy}\left(U_{1}\bar{V_{1}}+{\bar{U}}_{1}V_{1}\right)+\\ \lambda_{1}\left\{\begin{array}{c} 2\alpha^{2}\left(2U_{1}{\bar{U}}_{1}-U_{1}\bar{P_{1}}-{\bar{U}}_{1}P_{1}\right)+{U^{\prime }}_{1}{\bar{P}}^{\prime }{}_{1}-{\bar{U}}_{1}^{\prime }P_{1}^{\prime }\\ -i\alpha \left(2{\bar{U}}_{1}V_{1}-2{U^{\prime }}_{1}\bar{V_{1}}+\bar{V_{1}}{P^{\prime }}_{1}-V_{1}{\bar{P_{1}}}^{\prime }\right) \end{array}\right\} \end{array}$$

(24) $$V_{20}\left(\pm 1\right)=0,U_{20}\left(\pm 1\right)\pm \frac{1}{2}\left({\bar{U}}_{1}^{\prime }\left(\pm 1\right)+U_{1}^{\prime }\left(\pm 1\right)\right)=0.$$

Simple integration yields the solution of the above equations as

(25) $$V_{20}\left(y\right)=-\chi \left(P_{1}\bar{V_{1}}+\bar{P_{1}}V_{1}\right)+D_{1},$$

(26) $$U_{20}\left(y\right)=ReE\left(y\right)+D_{2}y+D_{3}$$

Here, E(y)=∬f(y)dydy,$\;D_{1}=0$, where $D_{2}$ and $D_{3}$ are calculated using the boundary condition given in Equation (24). Following Fung and Yih [44], the mean velocity perturbation function, the mean axial velocity and dimensionless net flow rate are expressed as

(27) $$G\left(y\right)=\frac{-200}{\alpha^{2}Re^{2}}\left(E\left(y\right)-E\left(1\right)\right),\langle u\rangle =\varepsilon^{2}U_{20}\left(y\right)\mathrm{and}\;\langle Q\rangle =\varepsilon^{2}\int_{0}^{1}U_{20}\left(y\right)dy$$

The quantity 〈*Q*〉 defined above is in dimensionless form and therefore independent of any units.

## 4. Discussion

This section aims to elaborate the significance of various parameters on the mean axial velocity, $D_{wall}$ dimensionless flow rate and perturbation function G(y). The emphasis has been given to the time relaxation parameter $\lambda_{1}$ both linear and convected Maxwell liquid models, compressibility paramater χ and the wave number α.

**Figure 2:**
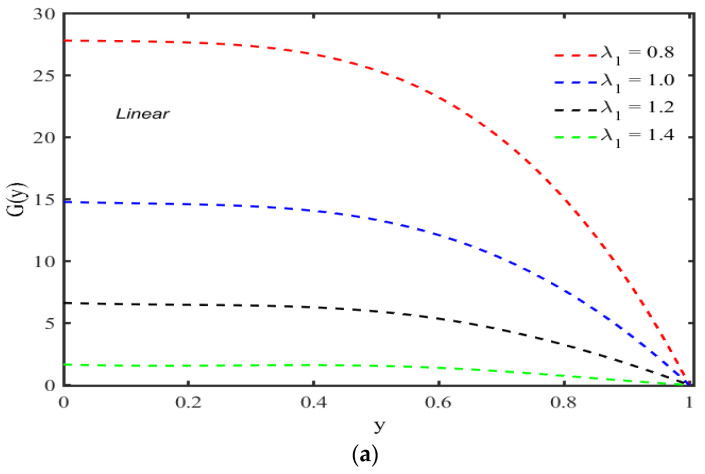

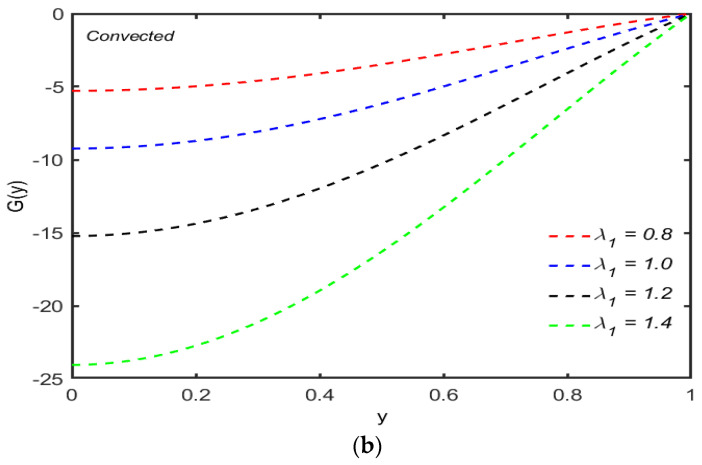
(**a**) Change in G(y) for several values of $\lambda_{1}$ with R=10,χ=0.3,α=0.5. (**b**) Change in G(y) for several values of $\lambda_{1}$ with R=10, χ=0.3,α=0.5.

The mean velocity perturbation function G(y) for Maxwell models are plotted in Figure 2a,b; these figures reveal that the perturbation function decreases by increasing the values of $\lambda_{1}$ in case of a linear model while increasing in case of a convected Maxwell model. Figure 2a also depicts that for small values of $\lambda_{1}$, the perturbation function is constant for y≤0.5, and by increasing the relaxation parameter, this region gradually decreases with increasing $\lambda_{1}$, but this indication cannot be seen through Figure 2b. Furthermore, the magnitude of the perturbation function at the center of the channel for the case of a linear model is greater in comparison with the convected model.

**Figure 3:**
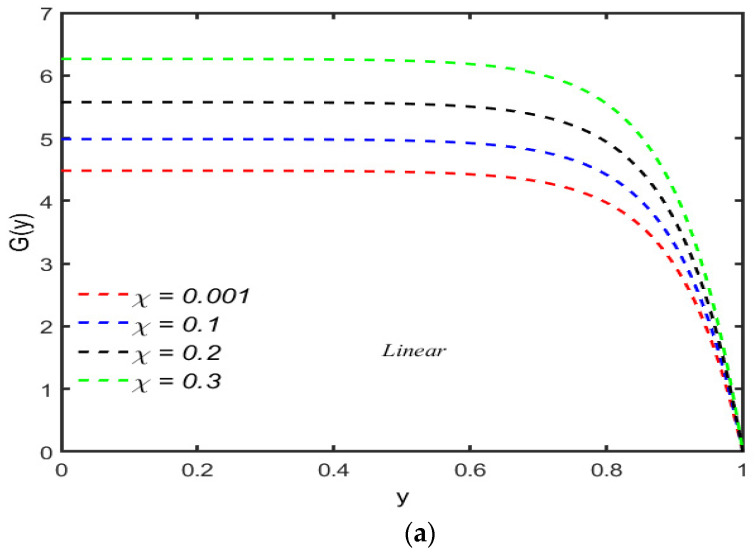

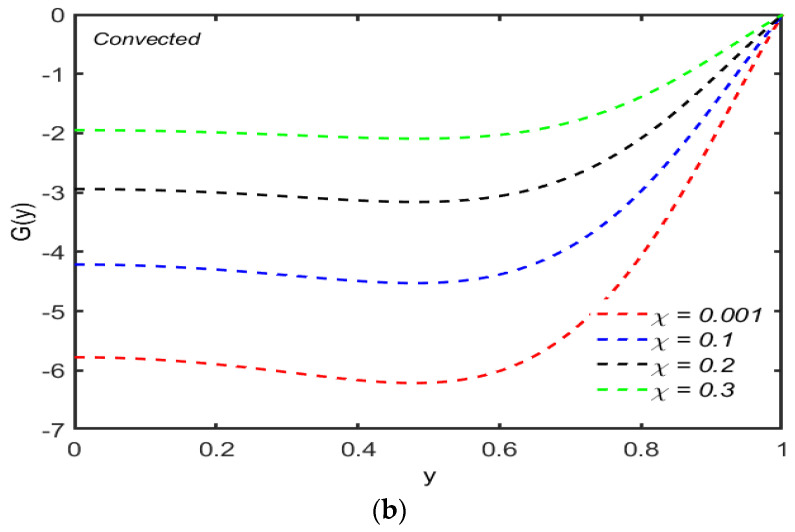
(**a**) Change in G(y) for χ with $R=100,\alpha =0.5,\lambda_{1}=0.5$. (**b**) Change in G(y) for χ with $R=100,\alpha =0.5,\lambda_{1}=0.5$.

**Figure 4:**
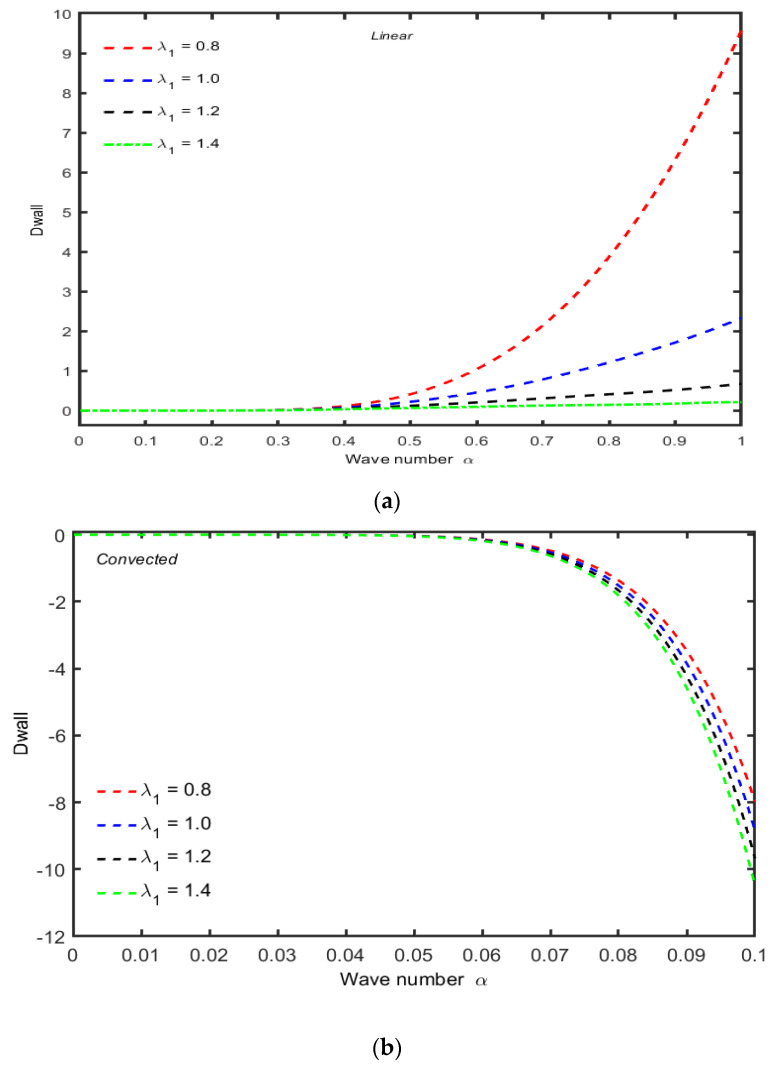
(**a**) Variation of $D_{wall}$ against the wave number α for several values of $\lambda_{1}$ when other parameters are R=50,χ=0.001. (**b**) Variation of $D_{wall}$ against α with $\lambda_{1}$ R=50,χ=0.001.

The perturbation function G(y) for the different values of the compressibility parameter χ is sketched in Figure 3a,b. Like the relaxation parameter, the compressibility parameter has opposite effects for linear and convected models. Figure 3 indicates that for the linear Maxwell model, up to a certain value of y, the perturbation function is constant for all the values of compressibility parameter χ and starts decreasing by increasing χ. The magnitude of the perturbation function at the heart of the channel is maximum when the analysis has been carried out by using the linear model. Figure 4 reflects that the perturbation function has an increasing trend for the compressibility of the fluid, but for small values of χ, there is a slight decrease in the G(y) against y up to a certain limit; moreover, the magnitude of G(y) when the convected model is used is less at the center of the channel. It is also concluded that for Newtonian fluid, the value of G(y) is constant for the half width of the channel. It is concluded that the viscoelasticity has great effects on the perturbation function.

The effects of time relaxation parameter $\lambda_{1}$ on the $D_{wall}$ against the wave number α are shown in Figure 4a,b; it is observed that there is almost no variation in $D_{wall}$ for small values of $\lambda_{1}$ for small wave numbers, and in case of a linear model, the amplitude of $D_{wall}$ starts increasing by increasing $\lambda_{1}$, and in case of a convected model, this variation starts almost for larger values. At the center of the channel, there is only a quantitative difference in the amplitude of $D_{wall}$. More precisely, similar to the perturbation function at the center of the channel, the amplitude of $D_{wall}$ is less in case of an upper convected Maxwell model. For the case of Newtonian fluid, the variation in $\;D_{wall}$ is higher than that of non-Newtonian material. For minimum values of the wave number, the variation in $D_{wall}$ increases for all values of $\lambda_{1}$ but decreases by increasing the values of $\;\lambda_{1}$ in the upper convected Maxwell fluid, which indicates that the viscoelasticity has significant effects on the variation in $D_{wall}$.

**Figure 5:**
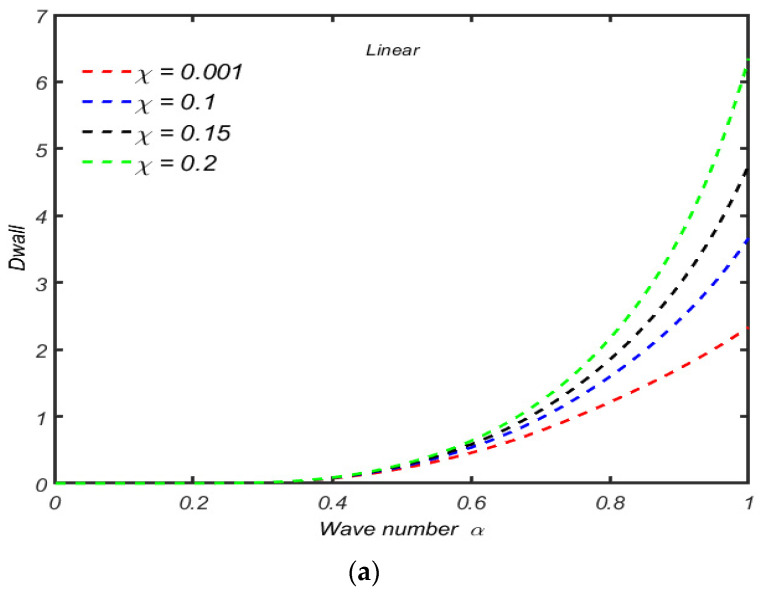

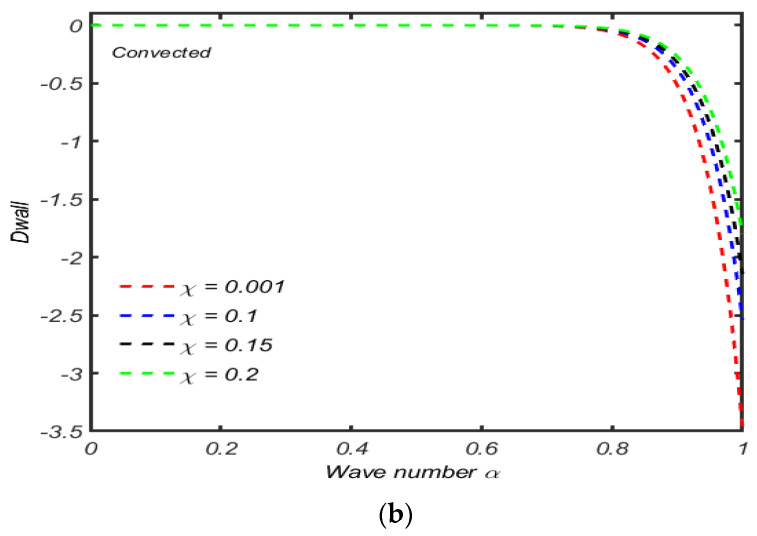
(**a**) Variation of $D_{wall}$ against α for χ with $R=10,\lambda_{1}=1$. (**b**) Variation of $D_{wall}$ against α for χ when other parameters are $R=10,\lambda_{1}=1$.

The impacts of compressibility on the $D_{wall}=U_{20}\left(1\right)$ are highlighted in Figure 5a,b. It is observed that for any value of α, the velocity at the boundary is increasing the compressibility of the fluid in case of a linear Maxwell model; also in this case, there is no indication of reverse flow. The variation of $D_{wall}$ with χ is depicted in Figure 5b. It is observed that the variation of $D_{wall}$ is negative for all values of χ and keeps on decreasing as χ increases—in this case, at the vicinity of the channel.

**Figure 6:**
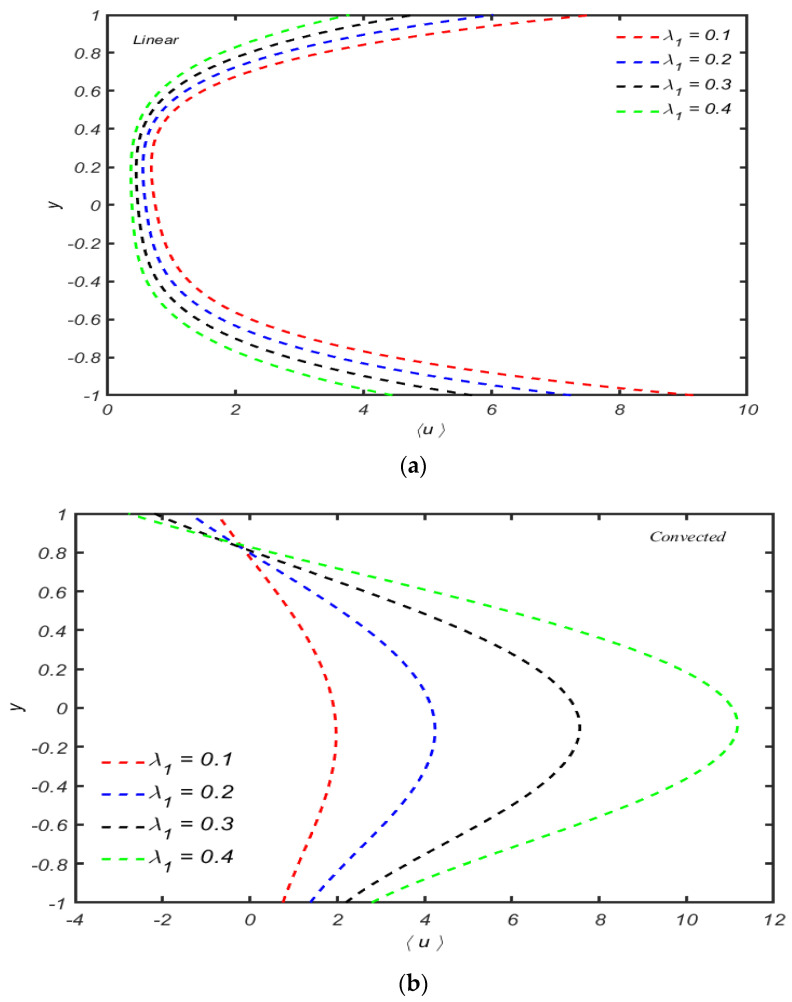
(**a**): The behavior of the mean axial velocity distribution for several values $\lambda_{1}$. When other parameters are R=10,α=0.5,χ=0.05. (**b**) Variation of the axial velocity distribution for several values $\lambda_{1}$ with R=10,α=0.5,χ=0.05.

The behavior of velocity distribution 〈u〉 versus y for distinct values of $\lambda_{1}$ is plotted in Figure 6a,b. These figures show that increasing the time relaxation parameter $\lambda_{1}$, the mean axial velocity increases for both cases (convected and linear). The magnitude of the mean axial velocity is maximum for the upper convected case. From Figure 6b, it is also observed that backward flow occurs near the walls.

**Figure 7:**
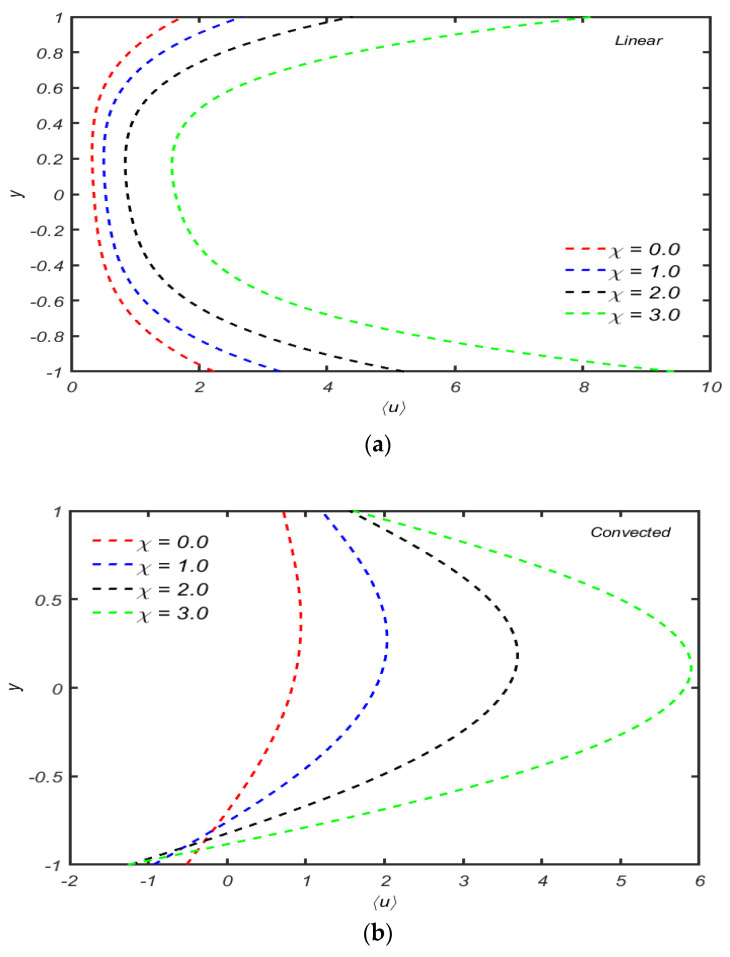
(**a**) Variation of mean axial velocity distribution for several values of χ with $R=10,\alpha =0.4,\;\;\lambda_{1}=0.5$. (**b**) Variation of mean axial velocity distribution for several values of χ when other parameters are $R=1,\alpha =0.4,\;\;\lambda_{1}=0.5$.

The response of velocity against y for compressibility parameters χ are shown in Figure 7a,b for the Maxwell linear model. The observations communicated that the mean flow distribution increases by increasing the compressibility parameter linear model, while the reverse behavior can be noted in the upper convected Maxwell fluid at the middle of the channel. The magnitude of mean axial velocity is maximum near the center and decreases.

**Figure 8:**
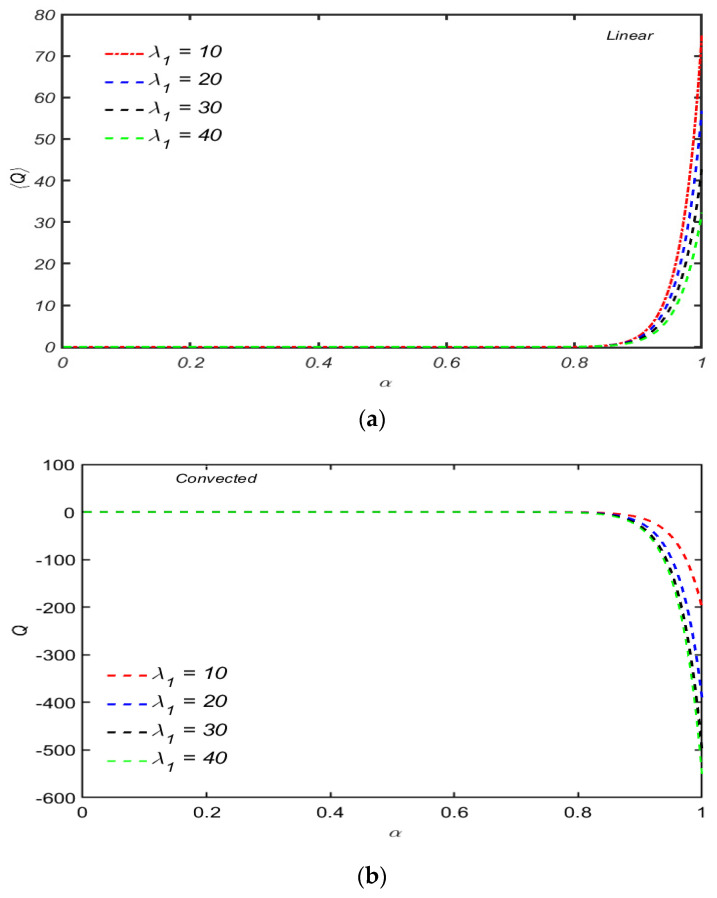
(**a**) The dimensionless net flow rate 〈Q〉 for several values of $\lambda_{1}$ when other parameters are $R=10^{4},\alpha =0.5$ and χ=0.3. (**b**) The dimensionless net flow rate 〈Q〉 for several values $\lambda_{1}$ when other parameters are $R=10^{4},\alpha =0.5$ and χ=0.3.

Figure 8a,b show the dimensionless flow rate for different values of $\lambda_{1}$ for both convected and linear Maxwell models against the wave number α. From both the figures, one can see that 0≤α≤0.75, which is almost the same and independent of the time relaxation parameter $\lambda_{1}$. The behavior of flow pattern for the Maxwell linear model is increasing, and the upper convected model shows decreasing behavior for α>0.75. Moreover, it is confirmed that a reverse flow pattern for the rate type material exists. These claims are quite opposite to the results reported by Abbasi et al. [22] when the same analysis was performed in an axisymmetric tube. This depicts that strong reverse flow has been induced by the propagation of a wave for the upper convected Maxwell model in contrast to the linear Maxwell model. Both the models provide the same flow trend behavior for small wave numbers and show opposite effects for larger values in case of small and large Reynolds numbers.

**Figure 9:**
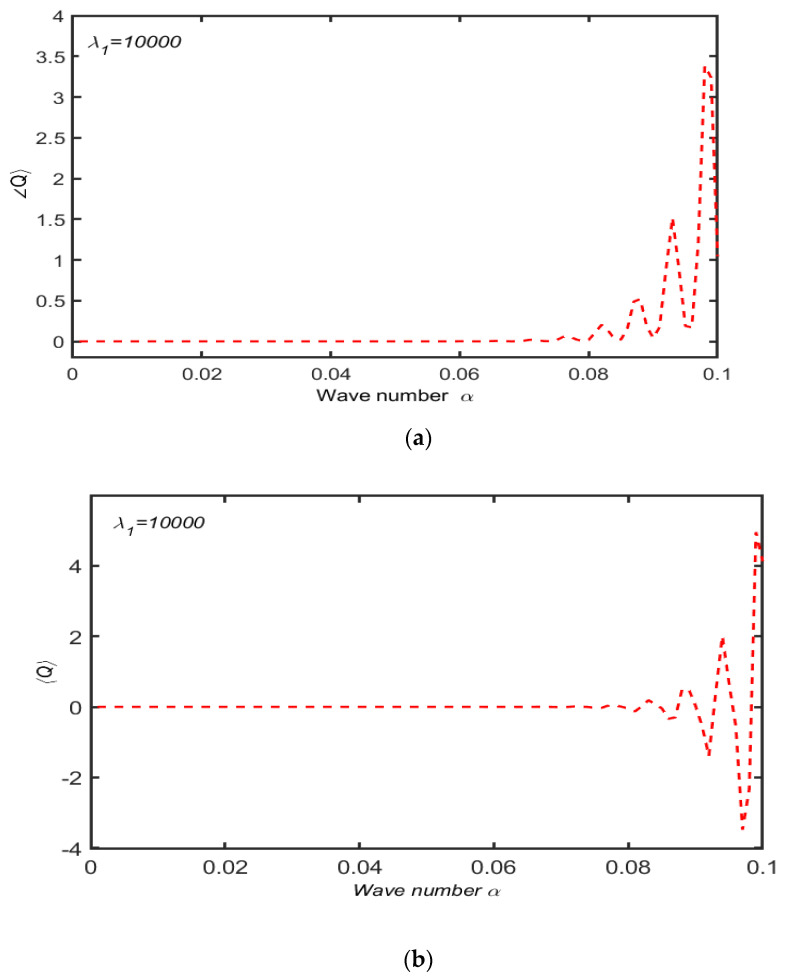
(**a**) Dimension-less net flow rate 〈Q〉 for linear model with $\lambda_{1}=10000$ when other parameters are $R=10^{4},\;\;\chi =0.6$. (**b**) Dimensionless net flow rate 〈Q〉 for convected model with $\lambda_{1}=10000$ when other parameters are $R=10^{4},\;\;\chi =0.6$.

Figure 9a,b show the flow rate for large values of time relaxation: parameter $\lambda_{1}=10000$ is plotted. Both figures indicate that the net flow rate is the same for both the models in a certain region 0≤α≪0.07 and oscillatory for α>0.08. The flow rate is positive in case of a linear model, while the negative values attained by the net flow rate for Maxwell fluid predict the possibility of back flow. A quantitative analysis of both the figures depicts that for the Maxwell upper convected model, the value of the mean flow rate is higher compared to the Maxwell linear model.

## 5. Main Findings

The flow generated by a peristaltic wave train in a microchannel is taken into account in this investigation. The constitutive equation of an upper convected Maxwell model is utilized. The solutions are obtained using a perturbation method; the axial velocity and net flow rate are obtained through numerical integration. A comparison between the linear and upper convected Maxwell material is presented. The main findings are summarized below:

- The impact of various parameters on the perturbation function G(y) reports contrary results between the linear and upper convected Maxwell fluid. It is observed that the $\lambda_{1},\chi$ Maxwell parameters have decreasing effects on G(y) for the linear model, while the $\lambda_{1},\chi$ Maxwell parameter have increasing effects on G(y) for the convected model.
- The effectiveness of different parameters on $D_{wall}$ with wave number α are different for both the upper convected as well as linear Maxwell fluid model.
- It is observed that the $\lambda_{1},\chi ,$ Maxwell parameters have increasing effects on $D_{wall}$ for the linear model, while the $\lambda_{1},\chi ,\;$ Maxwell parameters have decreasing effects on $D_{wall}$ for the convected Maxwell fluid model.
- The influences of different parameters on the net flow rate 〈Q〉 with wave number α and compressibility *χ* are different for linear and upper convected Maxwell fluid. It is observed that the $\lambda_{1}$ Maxwell parameter has increasing effects on the net flow rate 〈Q〉 for the linear model, while the $\lambda_{1},\chi ,$ Maxwell parameters have decreasing effects on 〈Q〉 for the convected model.

## Data Availability

All the data are available in the manuscript.

## Nomenclature

$A_{1}$	First Rivilin–Ericksen tensor	a	Amplitude of peristaltic wave
c	Wave speed	d	Dimensionless width of the channel
X	Compressibility parameter	S	Extra stress tensor
V	Velocity of field	〈u〉	Axial velocity
$\lambda_{1}$	Relaxation time	ρ	Density
Re	Reynold number	ϵ	Amplitude ratio
α	Wave parameter	〈Q〉	Flow rate
